# Annexin A2 Stabilizes Oncogenic JAG1 Intracellular Domain by Inhibiting Proteasomal Degradation in Glioblastoma Cells

**DOI:** 10.3390/ijms241914776

**Published:** 2023-09-30

**Authors:** Seok Won Ham, Jung Yun Kim, Sunyoung Seo, Nayoung Hong, Min Ji Park, Yoonji Kim, Junseok Jang, Sehyeon Park, Silvee Jisoo Lee, Jun-Kyum Kim, Eun-Jung Kim, Sung-Ok Kim, Sung-Chan Kim, Jong-Whi Park, Hyunggee Kim

**Affiliations:** 1 Department of Biotechnology, College of Life Sciences and Biotechnology, Korea University, Seoul 02841, Republic of Korea; sw-ham@medific.co.kr (S.W.H.); jyk00300@gmail.com (J.Y.K.); zpdlrmemr@naver.com (S.S.); nyhong0311@naver.com (N.H.); drannepark@naver.com (M.J.P.); yoonjikim0710@gmail.com (Y.K.); kkc4152@naver.com (J.J.); pshyeon61@korea.ac.kr (S.P.); silvee97@daum.net (S.J.L.); 2 Institute of Animal Molecular Biotechnology, Korea University, Seoul 02841, Republic of Korea; 3 MEDIFIC Inc., Hwaseong-si 18469, Republic of Korea; jk-kim@medific.co.kr (J.-K.K.); ej-kim@medific.co.kr (E.-J.K.); 4 Department of Biochemistry, College of Medicine, Hallym University, Chuncheon 24252, Republic of Korea; tjddhr4826@naver.com (S.-O.K.); biokim@hallym.ac.kr (S.-C.K.); 5 Department of Life Sciences, Gachon University, Incheon 21999, Republic of Korea

**Keywords:** JAG1, JICD1, ANXA2, Glioblastoma, cancer stem cell

## Abstract

Glioblastoma (GBM) is the most lethal brain cancer, causing inevitable deaths of patients owing to frequent relapses of cancer stem cells (CSCs). The significance of the NOTCH signaling pathway in CSCs has been well recognized; however, there is no NOTCH-selective treatment applicable to patients with GBM. We recently reported that Jagged1 (JAG1), a NOTCH ligand, drives a NOTCH receptor-independent signaling pathway via JAG1 intracellular domain (JICD1) as a crucial signal that renders CSC properties. Therefore, mechanisms regulating the JICD1 signaling pathway should be elucidated to further develop a selective therapeutic regimen. Here, we identified annexin A2 (ANXA2) as an essential modulator to stabilize intrinsically disordered JICD1. The binding of ANXA2 to JICD1 prevents the proteasomal degradation of JICD1 by heat shock protein-70/90 and carboxy-terminus of Hsc70 interacting protein E3 ligase. Furthermore, JICD1-driven propagation and tumor aggressiveness were inhibited by ANXA2 knockdown. Taken together, our findings show that ANXA2 maintains the function of the NOTCH receptor-independent JICD1 signaling pathway by stabilizing JICD1, and the targeted suppression of JICD1-driven CSC properties can be achieved by blocking its interaction with ANXA2.

## 1. Introduction

Glioblastoma (GBM) is the most lethal and common cancer of the central nervous system. Patients with GBM have a remarkably poor prognosis, with a median survival of 12–15 months and a 5-year survival rate of 5.1% [1]. The major cause of morbidity in patients with GBM is the limited availability of therapeutic options [2]. To date, anti-cancer therapy for GBM relies entirely on surgical bulk resection followed by concomitant temozolomide and radiation therapy, with prolonged survival of only by 2–3 months [3]. Therefore, it is necessary to develop effective therapeutic modalities for GBM.

Cancer initiation and propagation are dominated by a small subset of tumor-initiating cells called cancer stem cells (CSCs) [4]. Moreover, CSCs can differentiate into clones with diverse characteristics, resulting in intratumoral heterogeneity, which is a prominent feature of high-grade tumors [5]. However, there is no available anti-cancer therapy targeting CSCs [6]; therefore, understanding essential signaling pathways involved in the maintenance and survival of CSCs would enable the development of effective anti-cancer treatments.

The NOTCH signaling pathway is an essential regulator of CSC maintenance [7,8]. The canonical NOTCH pathway is triggered by ligand–receptor interactions and is highly regulated in a context-dependent manner. NOTCH undergoes serial cleavages mediated by A disintegrin and metalloprotease 10 (ADAM10)- or ADAM17/tumor necrosis factor α-converting enzyme and γ-secretase in the transmembrane domain, releasing NOTCH intracellular domain (NICD) from the plasma membrane (Supplementary Figure S1A) [7,8]. NICD then enters the nucleus to form a complex with centromere-binding protein 1/suppressor of hairless/lag-1 and mastermind-like protein, which directly regulate the expression of NOTCH downstream target genes [7,8].

Since the first clinical trial for T-cell acute lymphoblastic leukemia and acute myeloid leukemia in 2006, a variety of NOTCH-targeting drugs, such as γ-secretase inhibitors (GSIs) and antibodies, have been developed and evaluated for clinical applications [8,9]. However, NOTCH blockade using GSIs may result in unexpected adverse effects because GSIs not only inhibit the processing of NOTCH, but also affect the cleavage of other transmembrane proteins [10].

Notably, ICDs of NOTCH ligands, JAG (JAG1 and JAG2) and delta-like (DLL; DLL1, DLL3, and DLL4) families, can be released by the cleavage of ADAM10/17 and γ-secretase as well, under the regulation of various oncogenic signaling pathways (Supplementary Figure S1A) [10,11]. Notably, our previous study showed that JAG1 intracellular domain (JICD1) constitutes a transcriptional complex with DEAD-box helicase 17 (DDX17)/SMA- and MAD-related protein 3 (SMAD3)/TGFB-induced factor homeobox 2 (TGIF2) to promote the expression of the sex-determining region Y-box 2 (SOX2), which plays a crucial role in acquiring and maintaining CSC properties, including sphere-forming ability, invasiveness, and resistance to anti-cancer therapies in GBM [11].

JICD1 is an intrinsically disordered protein (IDP) that lacks a fixed or ordered three-dimensional structure [12]. IDPs adopt a fixed three-dimensional structure after binding to other proteins or macromolecules [12,13,14]. IDPs have unique functions, structures, interactions, and regulatory mechanisms that differ from structured proteins in many ways [14,15]. The abundance of IDPs in cells is strictly controlled for precise signal transduction in space and time, and the aberrant quantitative regulation or mutation of IDPs is associated with disease [13,15].

In the present study, we sought to identify the mechanism regulating the JICD1 signaling pathway. As a result, we discovered that annexin A2 (ANXA2) was an essential regulator of JICD1 stabilization by protecting against heat shock protein (HSP)-mediated proteasomal degradation.

## 2. Results

### 2.1. ANXA2 Binds to JICD1 to Regulate JICD1-Driven CSC Properties

To verify the molecular mechanism by which the JICD1 signaling pathway is regulated, we screened for proteins that bind to JICD1 using an unbiased approach. We assessed the results of immunoprecipitation (IP) against hemagglutinin (HA) in the LN18 GBM cell line expressing JICD1-HA-FLAG (LN18 JICD1-HA-FLAG) (Supplementary Figure S1A) [11]. The LN18 cell line, derived from the GBM tissue of a patient, has been generally used to model the GBM in vitro. The cell line harbors a point mutation in the TP53 gene and is deleted of the CDKN2A gene, recapitulating the human GBM having cellular context driven by such somatic alterations [16]. We identified 43 JICD1-binding proteins via mass spectrometry (MS) (Supplementary Figure S1B).

**Figure 1:**
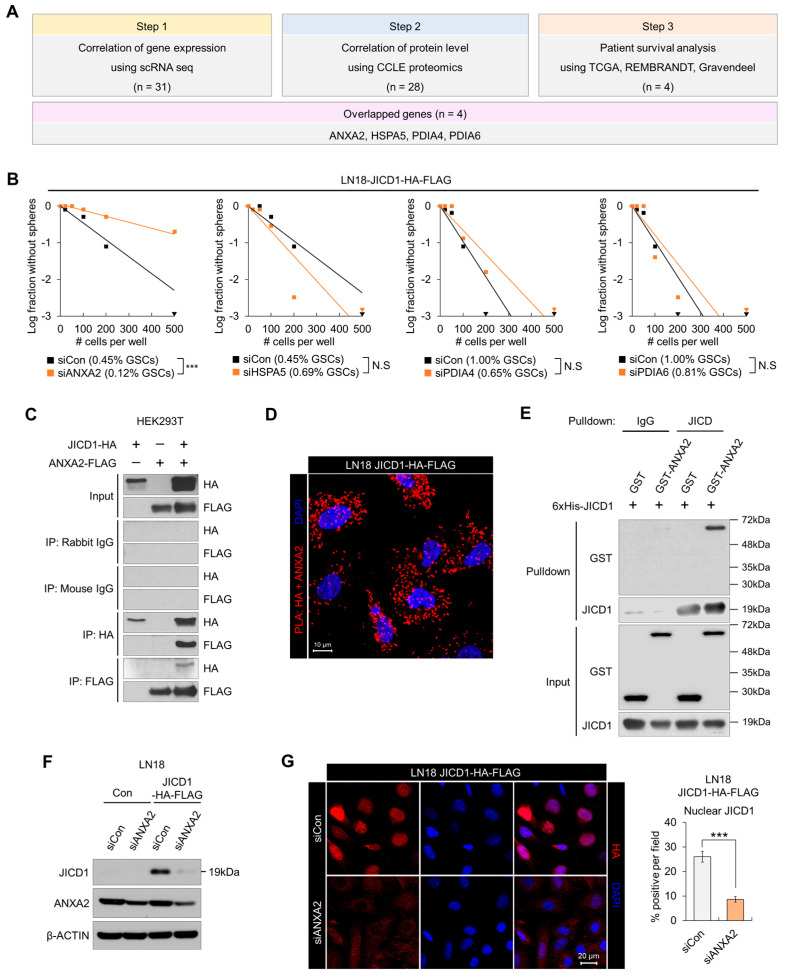
Annexin A2 (ANXA2) regulates JAG1 intracellular domain (JICD1)-driven CSC properties via interaction with JICD1. (**A**) The selection scheme of JICD1-binding protein using clinical databases. (**B**) Limiting dilution sphere-forming assay performed using LN18 cells expressing JICD1-HA-FLAG (LN18 JICD1-HA-FLAG) following the knockdown of genes encoding each JICD1-binding protein (ANXA2, protein disulfide-isomerase family A member 4 (PDIA4), PDIA6, and heat shock protein family A member 5 (HSPA5). *** *p* < 0.001. (**C**) Immunoprecipitation against HA and FLAG in HEK293T cells transduced with JICD1-HA and ANXA2-FLAG. (**D**) Microscopic images showing the results of proximity ligation assay against HA and ANXA2 in LN18 JICD1-HA-FLAG cells. Scale bar = 10 μm. (**E**) In vitro binding assay using the purified recombinant JICD1, and GST–ANXA2. The pull-down using IgG was used as a control. (**F**) Western blotting results showing that ANXA2 knockdown decreases JICD1 levels in LN18 cells. (**G**) Immunofluorescence staining showing that ANXA2 knockdown decreases nuclear JICD1 levels. Scale bar = 20 μm. *** *p* < 0.001.

Next, we conducted a bioinformatics analysis to select the JICD1-binding protein associated with the JICD1 signaling pathway in cancer. We calculated the correlation of gene expression between genes encoding each JICD1-binding protein and *JAG1* using a single cell RNA sequencing (scRNA-seq) database of patient-derived GBM samples [17]. Thirty-one genes were positively or negatively correlated with *JAG1* expression (Figure 1A). Similarly, we investigated the correlation of protein levels between JICD1-binding proteins and JAG1 in proteomics datasets from Cancer Cell Line Encyclopedia (CCLE). Twenty-eight proteins were significantly correlated with JAG1 expression (Figure 1A). We also conducted patient survival analysis using three different clinical datasets, the Cancer Genome Atlas (TCGA), the Repository for Molecular Brain Neoplasia Data (REMBRANDT), and Gravendeel, to identify the JICD1-binding protein that affects patient prognosis (Figure 1A). Four JICD1-binding proteins, ANXA2, heat shock protein family A member 5 (HSPA5), protein disulfide-isomerase family A member 4 (PDIA4), and PDIA6, were selected for all analyses.

To select the protein that affects JICD1-driven CSC properties, we performed limiting dilution assay (LDA) following the knockdown of each of the genes encoding the four JICD1-binding proteins in LN18 JICD1-HA-FLAG (Figure 1B). Only ANXA2 knockdown significantly decreased the sphere-forming ability (Figure 1B). ANXA2 knockdown also inhibited sphere formation in the JICD1-overexpressing cell lines A1207 and A172, and patient-derived 84NS Glioblastoma cancer stem cell (GSC) (Supplementary Figure S1C).

Immunofluorescence imaging revealed the co-localization of JICD1 and ANXA2 (Supplementary Figure S1D). Additionally, we confirmed the binding of JICD1 and ANXA2 by performing several protein-binding assays in both cellular and non-cellular formats. IP and proximity ligation assay (PLA) were performed using LN18 JICD1-HA-FLAG or transformed human embryonic kidney 293 (HEK293T) cells transduced with JICD1-HA and ANXA2-FLAG (Figure 1C,D and Supplementary Figure S1E). The IP results showed that JICD1 and ANXA2 bound to each other. Similarly, ANXA2-FLAG bound to C-terminal-HA-tagged JAG1, implying that ANXA2 has an affinity for the non-cleaved intracellular domain of JAG1 and JICD1 cleaved from JAG1 (Supplementary Figure S1F). To verify whether JICD1 directly binds to ANXA2, we performed in vitro binding assay (IVB) using purified recombinant proteins. GST-tagged ANXA2 was pulled down along with 6xHis-tagged JICD1 using an anti-JICD1 antibody (Figure 1E and Supplementary Figure S1G). Thus, ANXA2 binds to JICD1 and is a potential regulator of JICD1-driven CSC properties and GBM cell propagation.

ANXA2, a member of the annexin protein family, exists as a monomer or heterotetramer, and forms a complex with S100 calcium-binding protein A (S100A) 10 or S100A11, in which a dimer of S100A connects two ANXA2 monomers [18,19]. Previous findings have shown the function of ANXA2 in diverse biological processes, including the dynamic regulation of the cytoskeleton, activation of plasmin, and organization of exo- and endocytosis [20,21]. ANXA2 also accelerates the progression of various cancers; specifically, ANXA2 is highly expressed in GBM compared with that in low-grade glioma and promotes proliferation and invasion [22,23].

Because we found that ANXA2 binds to JICD1 and that its knockdown decreases the sphere-forming ability, we next investigated whether ANXA2 directly modulates the JICD1 signaling pathway. We knocked down ANXA2 expression using short interfering RNAs (siRNAs) and measured JICD1 levels. ANXA2 knockdown markedly decreased JICD1 levels in both LN18 and A1207 cells (Figure 1F,G and Supplementary Figure S1H,I).

Therefore, we sought to understand how ANXA2 knockdown decreases JICD1 levels. We measured *JAG1* mRNA expression following ANXA2 knockdown and found no notable changes compared with those found during the reduction in JICD1 levels (Figure 1F,G and Supplementary Figure S1J).

### 2.2. ANXA2 Inhibits Heat Shock Protein 70/90-Mediated Degradation of JICD1

**Figure 2:**
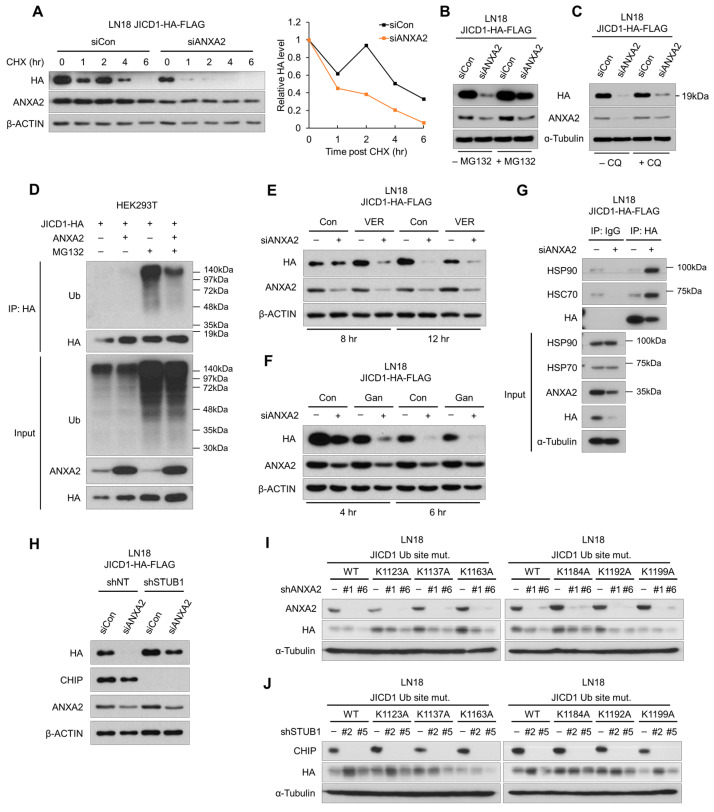
Lys^1184^ of JICD1 is ubiquitinated via heat shock protein (HSP)70/90-mediated degradation after ANXA2 knockdown. (**A**) Time-dependent changes in the level of JICD1-HA-FLAG following ANXA2 knockdown combined with cycloheximide treatment. (**B**) The protein level of JICD1-HA-FLAG following ANXA2 knockdown combined with MG132 treatment in LN18-JICD1-HA-FLAG cells. (**C**) The protein level of JICD1-HA-FLAG following ANXA2 knockdown combined with chloroquine (CQ) treatment in LN18 JICD1-HA-FLAG cells. (**D**) Immunoprecipitation against JICD1-HA to compare the ubiquitination of JICD1-HA with or without ANXA2 overexpression. (**E**,**F**) Levels of JICD1 in LN18 JICD1-HA-FLAG cells, following ANXA2 knockdown combined with (**E**) VER-155008 or (**F**) ganetespib. (**G**) Immunoprecipitation against HA to compare the binding with HSP70 and HSP90 after ANXA2 knockdown in LN18 JICD1-HA-FLAG cells. (**H**) Levels of JICD1 after ANXA2 knockdown, with or without concurrent knockdown of carboxy-terminus of Hsc70 interacting protein (CHIP) E3 ligase. (**I**) Levels of JICD1 with mutants in possible ubiquitination sites after ANXA2 knockdown. (**J**) Levels of JICD1 with mutants in possible ubiquitination sites, with or without knockdown of CHIP E3 ligase.

We estimated the difference in the stability of JICD1, following ANXA2 knockdown in the presence of cycloheximide (CHX), an inhibitor of ribosomal translation. When de novo protein synthesis was blocked, JICD1 decreased significantly faster, following ANXA2 knockdown in LN18 cells (Figure 2A). These results show that ANXA2 maintains the JICD1 signaling pathway by increasing the stability of JICD1.

To verify how ANXA2 inhibits JICD1 degradation, we blocked the protein degradation pathways in combination with ANXA2 knockdown. When treated with the proteasomal inhibitor MG132, JICD1 expression did not decrease after ANXA2 knockdown. However, the lysosomal inhibitor chloroquine (CQ) did not affect the reduction in JICD1 expression (Figure 2B,C and Supplementary Figure S2A). Therefore, we determined whether ANXA2 affected the ubiquitination of JICD1 to inhibit its degradation by the proteasome. We performed the IP of JICD1-HA using HEK293T cells, with or without ANXA2 overexpression and/or treatment with MG132. ANXA2 markedly inhibited the ubiquitination of JICD1 (Figure 2D). Taken together, our data suggest that ANXA2 inhibits the ubiquitin-mediated proteasomal degradation of JICD1.

Next, we sought to determine the mechanism of JICD1 ubiquitination, which ANXA2 inhibits. Interestingly, the JICD1-binding proteins identified by IP-MS included several HSPs that process misfolded proteins (Supplementary Figure S2B). HSPA1A, HSPA2, HSPA5, and HSPA8 (HSC70) belong to the HSP70 family, whereas HSP90AA1 and HSP90AB1 belong to the HSP90 family.

HSP70 and HSP90 family proteins are ATP-dependent chaperones that are essential for protein quality control [24]. First, a misfolded protein is recognized by HSP70/90 as a client by exposure to hydrophobic amino acids or unlinked thiol (-SH) [24,25]. The protein then undergoes triage selection, wherein its fate (whether to be re-used or degraded) is determined. The decision relies on the direct recognition of the irreparable folding of the protein and the indirect “timer” mechanism based on the dwell time in the chaperone [25]. The carboxy-terminus of Hsc70 interacting protein (CHIP; encoded by *STUB1*) participates as an E3 ubiquitin ligase to ubiquitinate the client protein that is to be degraded [24,25].

To confirm whether HSPs were involved in the degradation of JICD1 in the absence of ANXA2, we treated cells with the HSP70 inhibitor VER-155008 (VER) or the HSP90 inhibitor ganetespib (Gan) with or without ANXA2 knockdown. VER and Gan inhibited the refolding of misfolded proteins; therefore, the client protein underwent degradation pathway. Both VER and Gan treatments increased the rate of ANXA2 knockdown induced JICD1 degradation (Figure 2E,F).

We performed IP with or without ANXA2 knockdown to investigate whether ANXA2 affects the binding of HSPs to JICD1. HSC70/HSP90 showed remarkably increased binding to JICD1 following ANXA2 knockdown in LN18-JICD1-HA-FLAG (Figure 2G). Correspondingly, when CHIP E3 ligase was knocked down using short hairpin RNAs (shRNA), the level of JICD1 was not affected by ANXA2 knockdown (Figure 2H). Taken together, in the absence of ANXA2 binding, JICD1 undergoes HSP70- and HSP90-mediated degradation and is ubiquitinated by CHIP E3 ligase.

To search for predicted or previously reported ubiquitination sites on JICD1, we used UbPred (https://thetruth.ccs.neu.edu/ubpred, accessed on 1 June 2023) and Cell Signaling Technology (Danvers, MA, USA) (CST) PhosphoSite Plus (v6.7.1.1) (https://www.phosphosite.org, accessed on 1 June 2023). Both software packages suggested that JICD1 contains a lysine (Lys) residue that is accessible for ubiquitination (Supplementary Figure S2C,D). Considering the confidence of each lysine residue, we speculated six lysine residues of JICD1 (Lys^1123^, Lys^1137^, Lys^1163^, Lys^1184^, Lys^1192^, and Lys^1199^ in JAG1) to determine the ubiquitination site of JICD1 by CHIP E3 ligase. We compared the expression of JICD1 with or without point mutations at each lysine residue in HEK293T and LN18 cells. The expression of JICD1 was similar with or without point mutations in HEK293T cells. In LN18 cells, wild-type (WT) JICD1 and mutant JICD1 showed similar mRNA expression; however, the protein level of all mutant JICD1 was higher than that of WT JICD1 (Supplementary Figure S2E–G). In addition, mutants with the substituted amino acids Lys^1123^, Lys^1184^, Lys^1192^, and Lys^1199^ of JICD1 showed significantly higher protein levels (Supplementary Figure S2F).

To validate the amino acids of JICD1 that are essential for proteasomal degradation, we confirmed JICD1 protein levels harboring point mutations at each lysine residue in LN18 cells with ANXA2 or CHIP E3 ligase knockdown (Figure 2I,J). The inhibition of ANXA2 nevertheless mitigated the reduction in JICD1^K1184A^ and JICD1^K1199A^ levels (Figure 2I). When CHIP E3 ligase was knocked down, the stabilization of JICD1^K1137A^, JICD1^K1163A^, JICD1^K1184A^, and JICD1^K1192A^ was also inhibited (Figure 2J). Therefore, Lys^1184^ of JICD1 was verified as an amino acid that is ubiquitinated, contributing to stabilization by ANXA2, and Lys^1137^, Lys^1163^, and Lys^1192^ were also verified as amino acids that could be ubiquitinated.

### 2.3. JICD1 Interacts with the Annexin Repeat 4 Domain within ANXA2

**Figure 3:**
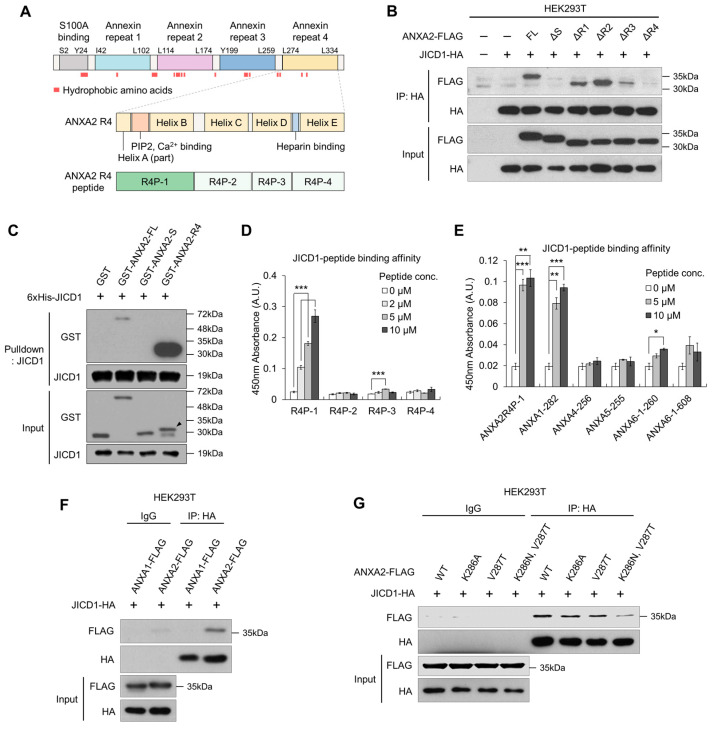
ANXA2 binds JICD1 via the ANXA2 repeat 4 (R4) domain. (**A**) A domain map of ANXA2 with the indication of hydrophobic amino acid residues. (**B**) Immunoprecipitation against JICD1-HA to compare the binding affinity between JICD1 and each deletion mutant of ANXA2. (**C**) In vitro binding assay using the purified recombinant JICD1 and GST-tagged ANXA2, S, and R4 domains. The black arrow indicates the band of interest. (**D**) JICD1-peptide binding assay using the ANXA2 R4 peptides. *** *p* < 0.001. (**E**) JICD1-peptide binding assay using the peptides having sequences of other annexin family proteins similar to R4P-1. * *p* < 0.05, ** *p* < 0.01, *** *p* < 0.001. (**F**) Immunoprecipitation against JICD1-HA using HEK293T cells to compare the JICD1-binding affinity of ANXA1 and ANXA2. (**G**) Immunoprecipitation against JICD1-HA using HEK293T cells expressing ANXA2 with mutants in Lys^286^ and Val^287^.

ANXA2 consists of five domains; one S100A-binding (S) domain and four annexin repeats (R1–4), and hydrophobic amino acid residues form a pocket on the opposite side of the calcium-binding surface (Figure 3A and Supplementary Figure S3A) [19]. To discover the JICD1 binding domain within ANXA2, we established a FLAG-tagged deletion mutant of ANXA2 and compared its binding affinity to JICD1. WT full-length ANXA2 and mutants lacking the R1, 2, and 3 domains (ΔR1, ΔR2, and ΔR3, respectively) could bind to JICD1. However, mutants lacking the S domain (ΔS) or R4 domain (ΔR4) did not bind to JICD1 (Figure 3B).

To identify the domain that directly interacts with JICD1, we then performed IVB using purified recombinant proteins. GST-tagged R4 showed a binding affinity for JICD1, whereas the S domain did not show such affinity (Figure 3C). Similarly, recombinant ANXA2 missing the S domain (ANXA2 ΔS) directly bound to JICD1, showing that JICD1 binds to the R4 domain of ANXA2 (Supplementary Figure S3B).

Next, we sought to identify the motif in the R4 domain essential for binding JICD1. We divided the R4 domain into four parts according to its secondary structure and synthesized peptides corresponding to the sequence of each part (R4P-1, -2, -3, and -4) (Figure 3A and Supplementary Figure S3C). Using these peptides, we designed an assay to measure the binding affinity of each peptide to JICD1 (Supplementary Figure S3D). In the binding assay, R4P-1, which is equivalent to Leu^274^–Ser^294^ in ANXA2, showed an affinity for JICD1 (Figure 3D).

We further identified several annexin family proteins with amino acid residues similar to those of R4P-1 using Protein Basic Local Alignment Search Tool (Protein BLAST). Among these, we selected amino acid residues with similarities to those of R4P-1 greater than 70% to synthesize the corresponding peptides (Supplementary Figure S3E). The peptide-binding assay revealed that only a peptide equivalent to Leu^282^–Ser^302^ of ANXA1 (ANXA1-282) had an affinity for JICD1, similar to that of R4P-1 (Figure 3E). However, ANXA1 did not bind to JICD1 (Figure 3F).

To validate an amino acid residue that critically renders the binding affinity to JICD1 in ANXA2, we clustered the sequences of the peptides, including R4P-1 and those similar to R4P-1. Considering the nature of amino acids constituting each peptide and their binding affinity to JICD1, we speculated that Lys^286^ and Val^287^ in ANXA2 (corresponding to Lys^394^ and Ala^395^ in ANXA1, respectively) would determine its affinity to JICD1 (Supplementary Figure S3F). We performed IP using ANXA2 harboring point mutations in each amino acid residue or both amino acid residues. The results showed that point mutations in both amino acid residues decreased the binding affinity to JICD1 (Figure 3G). Taken together, Leu^274^ to Ser^294^ in the R4 domain were primarily responsible for binding, and the two amino acids Lys^286^ and Val^287^ showed an affinity for JICD1 (Figure 3 and Supplementary Figure S3).

### 2.4. JICD1–ANXA2 Interaction Promotes Tumor Proliferation and Aggressiveness

**Figure 4:**
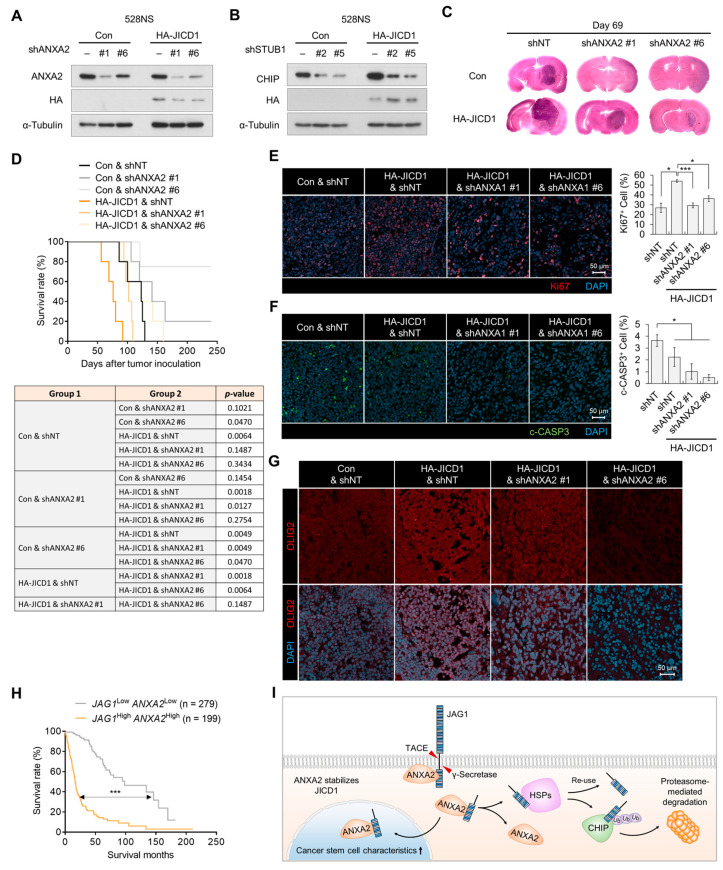
ANXA2 knockdown inhibits JICD1-driven cancer stem cell properties. (**A**) Levels of HA-JICD1 with or without ANXA2 knockdown in 528NS cells. (**B**) Levels of HA-JICD1 with or without CHIP knockdown in 528NS cells. (**C**) Representative images of hematoxylin and eosin staining. The time of mouse sacrifice is 69 days after tumor inoculation. (**D**) Kaplan–Meier survival plot showing survival of mice grafted with HA-JICD1 expressing 528NS cells with or without ANXA2 knockdown. (**E**,**F**) Immunofluorescence images for (**E**) Ki67 and (**F**) cleaved caspase3. Scale bar = 50 μm. The bar graphs represent the means ± standard error of the mean (SEM). * *p* < 0.05, *** *p* < 0.001. (**G**) Immunofluorescence images for OLIG2. Scale bar = 50 μm. (**H**) Survival time of patients with glioma according to JAG1 and ANXA2 expression. The patients were divided into two groups (*JAG1* high and *ANXA2* high versus *JAG1* low and *ANXA2* low) based on their mRNA expression (mean ± SEM). *p*-value was calculated with a log-rank (Mantel–Cox) test. *** *p* < 0.001. (**I**) A graphic summary showing the mechanism of JICD1 degradation in the absence of annexin A2-mediated stabilization and a possible therapeutic strategy to target the JICD1 signaling pathway.

To investigate the effect of ANXA2 knockdown on JICD1-induced aggressiveness, we transplanted JICD1 overexpressed 528NS GSCs with or without ANXA2 knockdown into the brains of BALB/c nude mice. Correspondingly, when ANXA2 was knocked down by an shRNA, the level of JICD1 decreased, and the knockdown of CHIP E3 ligase increased the level of JICD1 in 528NS GSCs (Figure 4A,B). JICD1 overexpression increased tumor propagation, whereas ANXA2 knockdown hindered tumor propagation 69 days after intracranial injection (Figure 4C, Supplementary Figure S4A,B). The shortened survival of mice by the overexpression of JICD1 was prolonged by the knockdown of ANXA2 (Figure 4D). JICD1 overexpression increased Ki67-positivity in the tumors, whereas ANXA2 knockdown decreased the positivity (Figure 4E). JICD1 overexpression decreased cleaved caspase3 (c-CASP3)-positive cells, whereas ANXA2 knockdown did not alter this ratio (Figure 4F). Consistent with the results of our previous study [11], the expression of SOX2, a JICD1 downstream gene, and OLIG2, a marker of GBM, was increased by JICD1 overexpression in 528NS tumors (Figure 4G and Supplementary Figure S4C). Notably, the knockdown of ANXA2 repressed the expression of SOX2 and OLIG2 (Figure 4G and Supplementary Figure S4C).

To investigate the clinical relevance of the JICD1–ANXA2 interaction, we analyzed the correlation between JAG1 and ANXA2 expression in TCGA GBMLGG data. The expression of both JAG1 and ANXA2 was higher in patients with high-grade glioma than in patients with grade II and III glioma and were positively correlated with each other (Supplementary Figure S5A,B). Likewise, patients with a higher expression of both JAG1 and ANXA2 had a significantly worse prognosis (Figure 4H). In addition, the expression of *STUB1* was negatively correlated with that of *JAG1* and *ANXA2* (Supplementary Figure S5B). Patients showing high *JAG1* expression and low *STUB1* expression had a worse prognosis than patients with low JAG1 expression and high *STUB1* expression (Supplementary Figure S5C). Taken together, our data show that ANXA2 stabilizes JICD1 by protecting it from HSP- and CHIP-mediated ubiquitination and degradation (Figure 4I).

## 3. Discussion

There are many approaches for treating cancer by suppressing the NOTCH signaling pathway. However, no NOTCH-targeted inhibitor is applicable for the treatment of GBM, owing to limitations such as low efficacy and the risk of adverse effects [26]. For instance, NOTCH blockade using GSIs also affects the processing of other transmembrane proteins, including insulin receptors, ErbB4, E-cadherin, and CD44 [10]. In addition, the consequences of NOTCH signaling are diverse depending on the genetic or epigenetic background and surrounding environmental factors, such as somatic mutations, including *EGFR* and *TP53*, and the ligand by which NOTCH is activated [27,28,29]. DLL1 and DLL4 activate the same NOTCH receptors and induce distinct cell fates during embryonic myogenesis [29]. Moreover, its ligand JAG1 drives independent signaling via JICD1 [11]. Therefore, precise therapeutic modalities targeting the JAG-NOTCH signaling pathway should be developed, considering the bidirectional signal transduction between JAG and NOTCH.

JAG-NOTCH signaling is a bidirectional signal transduction pathway [7]. In GBM, the expression of JAG1 is significantly correlated with aggressiveness rather than the expression of NOTCH receptors [11]. Unlike conventional NOTCH-targeting therapeutic inhibitors, a PPI inhibitor that blocks the interaction between JICD1 and ANXA2 is projected to specifically inhibit the signaling mechanism by the ligand JAG1. However, since JICD1 is an IDP lacking a three-dimensional structure, it is challenging to design a selective inhibitor that directly binds to JICD1 [12]. On the other hand, the structure of ANXA2 has been shown to contain a druggable pocket composed of hydrophobic amino acids, enabling the in silico modeling of drug candidates that potentially bind to the pocket and interfere with binding to JICD1 [30,31]. Therefore, the three-dimensional structure of the JICD1–ANXA2 complex should be determined to corroborate the structural evidence for the PPI between JICD1 and ANXA2 and to validate the mode of action and selectivity of the PPI inhibitor, which will be developed in the future.

In the present study, we elaborated on the pivotal function of ANXA2 in the JICD1 signaling pathway, suggesting the possibility of developing a novel CSC-targeted drug to treat GBM. Together with our previous findings, an in-depth study should be conducted to verify the involvement of ANXA2 in transcriptional regulation by JICD1 in complexes with DDX17, SMAD3, and TGIF2. The implications of JICD1 and ANXA2 should be investigated under distinct conditions, such as tumor vasculature or other types of cancer. Therefore, the tumor-initiating ability, invasiveness, and resistance to anti-cancer therapy imparted by JICD1 could be targeted.

## 4. Methods

### 4.1. Cell Lines and Cell Culture

The patient-derived GSCs 84NS (RRID: CVCL_C6J0) and 528NS (RRID: CVCL_C6IV) were kindly provided by Dr. Ichiro Nakano (University of Alabama, Birmingham, AL, USA). The cells were cultured in Dulbecco’s modified Eagle’s medium (DMEM)/F12 (GE Healthcare, Chicago, IL, USA) supplemented with 0.2% B27 (Invitrogen, Carlsbad, CA, USA), 20 ng/mL epidermal growth factor (EGF) (R&D Systems, Minneapolis, MN, USA), 20 ng/mL basic fibroblast growth factor (R&D Systems), 1% penicillin/streptomycin (GE Healthcare), 2 mmol/L L-glutamine (GE Healthcare), and 50 μg/mL gentamicin (Mediatech, Manassas, VA, USA). HEK293T (RRID: CVCL_0063), LN18 (RRID: CVCL_0392), A1207 (RRID: CVCL_8481), and A172 (RRID: CVCL_0131) cell lines were purchased from the American Type Culture Collection (Manassas, VA, USA). These cell lines were cultured in DMEM (GE Healthcare) supplemented with 10% fetal bovine serum (GE Healthcare), 1% penicillin/streptomycin, 2 mmol/L L-glutamine, and 50 μg/mL gentamicin. MG132, CQ, VER-155008, and CHX were purchased from Sigma-Aldrich (St. Louis, MO, USA). Ganetespib was purchased from Abmole Bioscience (Houston, TX, USA). Cell lines used in this study were regularly monitored to ensure the mycoplasma-free cells. In addition, all human cell lines were authenticated in 2019 using short tandem repeat profiling.

### 4.2. Overexpression and Knockdown of Genes

JICD1, JICD1-HA-FLAG, JICD1-HA, ANXA2, ANXA2-FLAG, JAG1-HA, ANXA2ΔS-FLAG, ANXA2ΔR1-FLAG, ANXA2ΔR2-FLAG, ANXA2ΔR3-FLAG, and ANXA2ΔR4-FLAG were cloned into the pcDNA3.1 vector for overexpression. The plasmids were transfected using LipoJet (SignaGen Laboratories, Frederick, MD, USA) for transient gene expression. To construct a stable cell line, microporation was performed using the Neon transfection system (Thermo Fisher Scientific, Waltham, MA, USA).

HA-JICD1, HA-JICD1^K1123A^, HA-JICD1^K1137A^, HA-JICD1^K1163A^, HA-JICD1^K1184A^, HA-JICD1^K1192A^, and HA-JICD1^K1199A^, were cloned into the pLL CMV BLAST vector for overexpression.

siRNAs targeting ANXA2 (SASI_Hs01_00246294), PDIA4) (SASI_Hs01_00081790), PDIA6 (SASI_Hs01_00124109), and HSPA5 (SASI_Hs01_00142880) were purchased from Sigma-Aldrich. siRNAs were delivered using ScreenFect (Wako, Osaka, Japan).

shRNAs targeting ANXA2 and CHIP were cloned into the pLKO.1 puro vector. A non-targeting shRNA (pLKO.1 shNT puro; Sigma-Aldrich) was used as a control. To produce lentiviruses, each expression vector was transfected into HEK293T cells with the second-generation lentiviral packaging plasmids pdR8.91 and pVSV g using LipoJet. Twenty-four hours after transfection, the culture medium was harvested, incubated with a Lenti-X concentrator (Clontech Laboratories, Mountain View, CA, USA), and centrifuged to obtain concentrated lentiviruses. The cells were infected with the lentiviruses in the presence of 6 μg/mL polybrene (Sigma-Aldrich, St. Louis, MO, USA) for 24 h.

### 4.3. LDA

The cells were seeded in a 96-well culture plate with serial dilution (Corning, Corning, NY, USA). The number of wells without spheres (having a diameter that exceeds 50 μm) was counted to estimate the frequency of CSCs, using Extreme Limiting Dilution Analysis (ELDA) software (WEHI Bioinformatics, Victoria, Australia).

### 4.4. Antibodies

The following antibodies were used in this study: JAG1 (C-terminal epitope, 70109; Cell Signaling Technology, Danvers, MA, USA), JAG1 (C-terminal epitope, PA5-72843; Thermo Fisher Scientific), ANXA2 (ab41803; Abcam, Cambridge, UK), rabbit IgG isotype control (10500C; Thermo Fisher Scientific), HA (3724; Cell Signaling Technology), HA (H9658, Sigma-Aldrich), FLAG (F7425; Sigma-Aldrich), β-ACTIN (sc-47778; Santa Cruz Biotechnology, Dallas, TX, USA), α-TUBULIN (T6199; Sigma-Aldrich), LMNB1 (ab16048; Abcam), Ubiquitin (3936; Cell Signaling Technology), HSP90 (PA3-013; Thermo Fisher Scientific), HSC70 (sc-7298; Santa Cruz Biotechnology), CHIP (2080; Cell Signaling Technology), glutathione-S-transferase (GST) (sc-138; Santa Cruz Biotechnology), Alexa 488 anti-mouse IgG (Invitrogen), and Alexa 594 anti-rabbit IgG (Invitrogen).

### 4.5. IP

Protein lysates were prepared using an IP lysis buffer (Thermo Fisher Scientific) and the lysates were pre-washed with protein A/G agarose beads (Thermo Fisher Scientific). The protein lysates were incubated overnight with an antibody against the bait protein. Antibody-bound proteins were precipitated using 3% bovine serum albumin (BSA)-blocked protein A/G agarose beads, and the beads were washed with the IP lysis buffer. Bead-bound proteins were eluted with 2-mercaptoethanol (Sigma-Aldrich)-added 2× lithium dodecyl sulfate (LDS, Invitrogen).

### 4.6. Western Blotting

Protein extracts from whole-cell lysates were prepared using a radioimmunoprecipitation assay lysis buffer (LPS Solution, Daejeon, Republic of Korea) containing 1 mM β-glycerophosphate, 2.5 mM sodium pyrophosphate, 1 mM NaF, 1 mM Na_3_VO_4_, and a protease inhibitor (Roche, Basel, Switzerland). Proteins were quantified via the Bradford assay (Bio-Rad, Hercules, CA, USA) according to the manufacturer’s protocol. Protein extracts were subjected to sodium dodecyl sulfate–polyacrylamide gel electrophoresis and transferred onto polyvinylidene difluoride membranes (Merck, Darmstadt, Germany). The membranes were blocked with 5% non-fat skim milk and incubated with primary antibodies. After incubation with horseradish peroxidase (HRP)-conjugated secondary antibodies, the target proteins were visualized using the SuperSignal West Pico chemiluminescent substrate (Elpis Biotech, Daejeon, Republic of Korea).

### 4.7. Immunofluorescence Imaging

The cells were fixed with a 4% paraformaldehyde solution for 10 min and washed with phosphate-buffered saline (PBS). The cells were blocked with 3% BSA for 1 h and incubated with primary antibodies overnight. The cells were then incubated with fluorescence-conjugated secondary antibodies and 4′,6-diamidino-2-phenylindole (Sigma-Aldrich). Images were obtained using an LSM800 confocal microscope (Carl Zeiss, Oberkochen, Germany).

### 4.8. PLA

PLA was performed to detect JICD1–ANXA2 binding using the Duolink PLA kit (Sigma-Aldrich) according to the manufacturer’s instructions. 

### 4.9. Purification of the Recombinant Proteins

JICD1 and ANXA2 were cloned into the pET28a and pGEX4T expression vectors for 6xHis and GST tagging, respectively. The expression vector was transduced into BL21-competent cells. 6×His-tagged proteins were purified using a lysis buffer (50 mM Na_2_HPO_4_, 50 mM NaH_2_PO_4_, 300 mM NaCl, 20 mM imidazole, and 0.1 mM PMSF; pH 8.0), elution buffer (50 mM Na_2_HPO_4_, 50 mM NaH_2_PO_4_, 300 mM NaCl, and 250 mM imidazole; pH 8.0), and Ni-NTA agarose beads (Qiagen, Hilden, Germany). GST-tagged proteins were purified using a lysis buffer (0.1 mM PMSF in PBS), elution buffer (10 mM glutathione in PBS), and GSH Sepharose beads (GE Healthcare). 

### 4.10. IVB

Purified recombinant proteins were incubated overnight with the IgG control or antibody in a binding buffer (50 mM Tris, 300 mM NaCl, 2 mM EDTA, 1 mM DTT, 0.1% NP-40, and 5 mg/mL BSA; pH 7.5). Antibody-bound proteins were precipitated using 3% BSA-blocked protein A/G agarose beads and washed with a washing buffer (50 mM Tris, 300 mM NaCl, 1 mM EDTA, 1 mM DTT, and 0.1% Tween-20; pH 7.5). Bead-bound proteins were eluted with 2-mercaptoethanol-added 2X LDS.

### 4.11. Quantitative Reverse Transcription Polymerase Chain Reaction 

Complementary DNA (cDNA) was synthesized from the extracted RNA using RevertAid First-Strand cDNA Synthesis Kit (Thermo Fisher Scientific). Quantitative polymerase chain reaction analysis was conducted using the CFX Connect real-time PCR detection system (Bio-Rad, Hercules, CA, USA) and SYBR Premix Ex Taq (Takara, Shiga, Japan) according to the manufacturer’s instructions. Further, 18S ribosomal RNA (*RN18S*) was used as a housekeeping control. The primer sequences were as follows: *JAG1* forward, 5′-GCTTGGATCTGTTGCTTGGT-3′, *JAG1* reverse, 5′-TGGGCACTTTCCAAGTCTCT-3′, *SOX2* forward, 5′-CAAGATGCACAACTCGGAGA-3′, *SOX2* reverse, 5′-CGGGGCCCGTATTTATAATC-3′, *RN18S* forward, 5′-CAGCCACCCGAGATTGAGCA-3′, *RN18S* reverse, 5′-TAGTAGCGACGGGCGGTGTG-3′.

### 4.12. JICD1-Peptide Binding Assay

Biotin-conjugated custom peptides, with sequences equivalent to those of ANXA2R4 and other annexin family proteins, were purchased from Bionics (Seoul, Republic of Korea). Further, 6xHis-JICD1 was added to a Pierce Ni-coated 96-well plate (Thermo Fisher Scientific) in a binding buffer (1% BSA in PBS). After washing with PBS containing 0.05% Tween-20, the peptides were added and incubated for 1 h. Peptides bound to JICD1 were detected using streptavidin–HRP (Invitrogen) and TMB substrate (Thermo Fisher Scientific). A Ni-coated plate without a coating of JICD1 was used as a negative control.

### 4.13. Peptide Sequence Clustering

The clusterization of the peptide sequence was performed using the Clustal Omega (v1.2.4) software (EMBL-EBL, Cambridgeshire, UK). 

### 4.14. Protein Structure Presentation

The protein structure of ANXA2 (PDB: 1XJL) was visualized using PyMol (Schrödinger, NY, USA).

### 4.15. Intracranial Xenograft Model

528NS-control and 528NS-HA-JICD1 cells transduced with shANXA2 were harvested and washed with PBS. Cell viability was determined using the trypan blue exclusion method. Single-cell suspensions with >90% viability were used for in vivo experiments. The cells (1 × 10^5^ cells/3 μL PBS) were stereotactically injected into the left striatum of 5-week-old BALB/c nu/nu mice (coordinates relative to the bregma: medial-lateral +2 mm and dorsal-ventral −3 mm). To compare tumor histology, all mice were sacrificed simultaneously when the second mouse showed neurological symptoms. To compare the aggressive phenotype after ANXA2 knockdown, brains were removed at the time of death. Kaplan–Meier survival was determined within 240 days.

### 4.16. Hematoxylin–Eosin Staining

To compare tumorigenicity, the samples were immersed in hematoxylin (Cat. #1.05174.0500; Merck) 10 times and rinsed with tap water and treated with eosin (Cat. #MA0101015; BBC Biochemical, Seoul, Republic of Korea) for 6 min, followed by dehydration in xylene. Finally, the slides were mounted using a mounting solution (Cat. #SP15-100; Thermo Fisher Scientific).

### 4.17. In Silico Analysis

To select the JICD1-binding protein associated with the JICD1 signaling pathway, common genes among the extracted genes were selected through the following three analyses. First, genes with positive or negative correlation with the gene expression of *JAG1* were corrected using a scRNA-seq database from patient-derived GBM samples (*p* < 0.05) [17]. Next, the JICD1-binding proteins correlated with the protein level of JAG1 were selected from proteomics datasets from CCLE (*p* < 0.05) [32]. Finally, genes that affect the overall survival of patients were selected through patient prognosis analysis using the following three datasets; TCGA, REMBRANDT, and Gravendeel (Log-rank *p* < 0.05 in all datasets) [33,34,35].

To verify the clinical relevance of the JICD1 stabilization mechanism, the mRNA expression of patients from the TCGA GBMLGG database generated by TCGA Research Network (https://www.cancer.gov/tcga, accessed on 1 January 2016) was used. High- and low-expression groups were separated based on their mRNA expression (mean ± standard error of the mean (SEM)).

### 4.18. Statistics

All data from the experiments, shown as bar graphs, are presented as the mean ± SEM. Data were analyzed using two-tailed Student’s *t*-tests (* *p* < 0.05; ** *p* < 0.01; *** *p* < 0.001). The Pearson product–moment correlation coefficient (*r*) was calculated using Microsoft Excel.

## Data Availability

All data and available upon request to the corresponding author.

## Supplementary Materials

The following supporting information can be downloaded at: https://www.mdpi.com/article/10.3390/ijms241914776/s1.

## References

[1] Ostrom Q.T., Price M., Neff C., Cioffi G., Waite K.A., Kruchko C., Barnholtz-Sloan J.S. (2022). CBTRUS Statistical Report: Primary Brain and Other Central Nervous System Tumors Diagnosed in the United States in 2015–2019. Neuro-Oncol..

[2] Lukas R.V., Wainwright D.A., Ladomersky E., Sachdev S., Sonabend A.M., Stupp R. (2019). Newly Diagnosed Glioblastoma: A Review on Clinical Management. Oncology.

[3] Keime-Guibert F., Chinot O., Taillandier L., Cartalat-Carel S., Frenay M., Kantor G., Guillamo J.S., Jadaud E., Colin P., Bondiau P.Y. (2007). Radiotherapy for glioblastoma in the elderly. N. Engl. J. Med..

[4] Reya T., Morrison S.J., Clarke M.F., Weissman I.L. (2001). Stem cells, cancer, and cancer stem cells. Nature.

[5] Eun K., Ham S.W., Kim H. (2017). Cancer stem cell heterogeneity: Origin and new perspectives on CSC targeting. BMB Rep..

[6] Prager B.C., Bhargava S., Mahadev V., Hubert C.G., Rich J.N. (2020). Glioblastoma Stem Cells: Driving Resilience through Chaos. Trends Cancer.

[7] Bray S.J. (2016). Notch signalling in context. Nat. Rev. Mol. Cell. Biol..

[8] Nowell C.S., Radtke F. (2017). Notch as a tumour suppressor. Nat. Rev. Cancer.

[9] Moore G., Annett S., McClements L., Robson T. (2020). Top Notch Targeting Strategies in Cancer: A Detailed Overview of Recent Insights and Current Perspectives. Cells.

[10] LaVoie M.J., Selkoe D.J. (2003). The Notch ligands, Jagged and Delta, are sequentially processed by alpha-secretase and presenilin/gamma-secretase and release signaling fragments. J. Biol. Chem..

[11] Kim E.J., Kim J.Y., Kim S.O., Hong N., Choi S.H., Park M.G., Jang J., Ham S.W., Seo S., Lee S.Y. (2022). The oncogenic JAG1 intracellular domain is a transcriptional cofactor that acts in concert with DDX17/SMAD3/TGIF2. Cell Rep..

[12] De Biasio A., Guarnaccia C., Popovic M., Uversky V.N., Pintar A., Pongor S. (2008). Prevalence of intrinsic disorder in the intracellular region of human single-pass type I proteins: The case of the notch ligand Delta-4. J. Proteome Res..

[13] Dunker A.K., Lawson J.D., Brown C.J., Williams R.M., Romero P., Oh J.S., Oldfield C.J., Campen A.M., Ratliff C.M., Hipps K.W. (2001). Intrinsically disordered protein. J. Mol. Graph. Model..

[14] Van der Lee R., Buljan M., Lang B., Weatheritt R.J., Daughdrill G.W., Dunker A.K., Fuxreiter M., Gough J., Gsponer J., Jones D.T. (2014). Classification of intrinsically disordered regions and proteins. Chem. Rev..

[15] Wright P.E., Dyson H.J. (2015). Intrinsically disordered proteins in cellular signalling and regulation. Nat. Rev. Mol. Cell Biol..

[16] Von Deimling A., Louis D.N., von Ammon K., Petersen I., Wiestler O.D., Seizinger B.R. (1992). Evidence for a tumor suppressor gene on chromosome 19q associated with human astrocytomas, oligodendrogliomas, and mixed gliomas. Cancer Res..

[17] Darmanis S., Sloan S.A., Croote D., Mignardi M., Chernikova S., Samghababi P., Zhang Y., Neff N., Kowarsky M., Caneda C. (2017). Single-Cell RNA-Seq Analysis of Infiltrating Neoplastic Cells at the Migrating Front of Human Glioblastoma. Cell Rep..

[18] Bharadwaj A., Bydoun M., Holloway R., Waisman D. (2013). Annexin A2 heterotetramer: Structure and function. Int. J. Mol. Sci..

[19] Gerke V., Creutz C.E., Moss S.E. (2005). Annexins: Linking Ca^2+^ signalling to membrane dynamics. Nat. Rev. Mol. Cell Biol..

[20] Morel E., Gruenberg J. (2009). Annexin A2 binding to endosomes and functions in endosomal transport are regulated by tyrosine 23 phosphorylation. J. Biol. Chem..

[21] Kim J., Hajjar K.A. (2002). Annexin II: A plasminogen-plasminogen activator co-receptor. Front. Biosci..

[22] Wang C.Y., Lin C.F. (2014). Annexin A2: Its molecular regulation and cellular expression in cancer development. Dis. Markers.

[23] Chen L., Lin L., Xian N., Zheng Z. (2019). Annexin A2 regulates glioma cell proliferation through the STAT3-cyclin D1 pathway. Oncol. Rep..

[24] Buchberger A., Bukau B., Sommer T. (2010). Protein quality control in the cytosol and the endoplasmic reticulum: Brothers in arms. Mol. Cell..

[25] Arndt V., Rogon C., Höhfeld J. (2007). To be, or not to be–Molecular chaperones in protein degradation. Cell. Mol. Life Sci..

[26] D’Amico M., De Amicis F. (2022). Aberrant Notch signaling in gliomas: A potential landscape of actionable converging targets for combination approach in therapies resistance. Cancer Drug Resist..

[27] Unger F.T., Witte I., David K.A. (2015). Prediction of individual response to anticancer therapy: Historical and future perspectives. Cell. Mol. Life Sci..

[28] Hoadley K.A., Yau C., Wolf D.M., Cherniack A.D., Tamborero D., Ng S., Leiserson M.D.M., Niu B., McLellan M.D., Uzunangelov V. (2014). Multiplatform analysis of 12 cancer types reveals molecular classification within and across tissues of origin. Cell.

[29] Nandagopal N., Santat L.A., LeBon L., Sprinzak D., Bronner M.E., Elowitz M.B. (2018). Dynamic Ligand Discrimination in the Notch Signaling Pathway. Cell.

[30] Lavecchia A., Di Giovanni C. (2013). Virtual screening strategies in drug discovery: A critical review. Curr. Med. Chem..

[31] Zinzalla G., Thurston D.E. (2009). Targeting protein-protein interactions for therapeutic intervention: A challenge for the future. Future Med. Chem..

[32] Nusinow D.P., Szpyt J., Ghandi M., Rose C.M., McDonald E.R., Kalocsay M., Jané-Valbuena J., Gelfand E., Schweppe D.K., Jedrychowski M. (2020). Quantitative Proteomics of the Cancer Cell Line Encyclopedia. Cell.

[33] Madhavan S., Zenklusen J.C., Kotliarov Y., Sahni H., Fine H.A., Buetow K. (2009). Rembrandt: Helping personalized medicine become a reality through integrative translational research. Mol. Cancer Res..

[34] Gravendeel L.A., Kouwenhoven M.C., Gevaert O., de Rooi J.J., Stubbs A.P., Duijm J.E., Daemen A., Bleeker F.E., Bralten L.B., Kloosterhof N.K. (2009). Intrinsic gene expression profiles of gliomas are a better predictor of survival than histology. Cancer Res..

[35] Ceccarelli M., Barthel F.P., Malta T.M., Sabedot T.S., Salama S.R., Murray B.A., Morozova O., Newton Y., Radenbaugh A., Pagnotta S.M. (2016). Molecular Profiling Reveals Biologically Discrete Subsets and Pathways of Progression in Diffuse Glioma. Cell.

