# Supplementary material for: Annexin A2 Stabilizes Oncogenic JAG1 Intracellular Domain by Inhibiting Proteasomal Degradation in Glioblastoma Cells
Source: Int J Mol Sci. 2023 Sep 30;24(19):14776. doi: 10.3390/ijms241914776 (PMC10573421; doi:10.3390/ijms241914776)
Supplement: Supplementary file 1 [file ijms-24-14776-s001.zip › ijms-2628105-supplementary.pdf]

Annexin A2 Stabilizes Oncogenic JAG1 Intracellular Domain by Inhibiting Proteasomal Degradation in Glioblastoma Cells

Seok Won Ham[1,2,3,†], Jung Yun Kim[1,2,†], Sunyoung Seo[1,2], Nayoung Hong[1,2], Min Ji Park[1,2], Yoonji Kim[1,2], Junseok Jang[1,2], Sehyeon Park[1,2], Silvee Jisoo Lee[1,2], Jun-Kyum Kim[3], Eun-Jung Kim[3], Sung-Ok Kim[4], Sung-Chan Kim[4], Jong-Whi Park[5,*], and Hyunggee Kim[1,2,*]

[1]Department of Biotechnology, College of Life Sciences and Biotechnology, Korea University, Seoul 02841, Republic of Korea

[2]Institute of Animal Molecular Biotechnology, Korea University, Seoul 02841, Republic of Korea

[3]MEDIFIC Inc., Hwaseong-si, Gyeonggi-do 18469, Republic of Korea

[4]Department of Biochemistry, College of Medicine, Hallym University, Chuncheon 24252, Republic of Korea

[5]Department of Life Sciences, Gachon University, Incheon 21999, Republic of Korea

[†]These authors contributed equally to this work.

[*]**Correspondence**: Hyunggee Kim (e-mail) hg-kim@korea.ac.kr

(Telephone) +82-02-3290-3059

Jong-Whi Park (e-mail) jpark@gachon.ac.kr

(Telephone) +82-032-899-6115

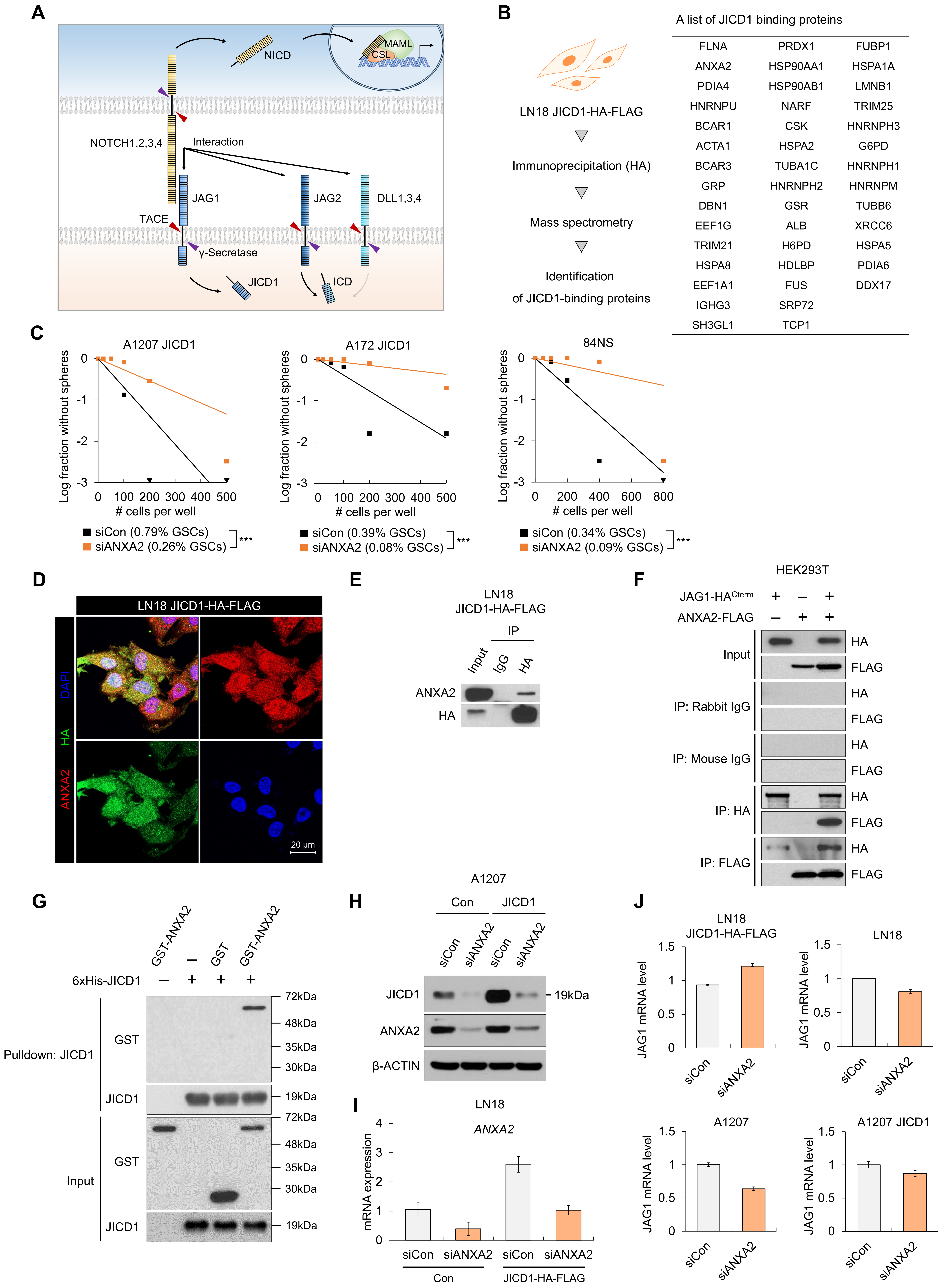
A
NICD
MAML
CSL
NOTCH1,2,3,4
Interaction
JAG1
JAG2
DLL1,3,4
TACE
γ-Secretase
JICD1
ICD
B
LN18 JICD1-HA-FLAG
Immunoprecipitation (HA)
Mass spectrometry
Identification
of JICD1-binding proteins
A list of JICD1 binding proteins
FLNA PRDX1 FUBP1
ANXA2 HSP90AA1 HSPA1A
PDIA4 HSP90AB1 LMNB1
HNRNPU NARF TRIM25
BCAR1 CSK HNRNPH3
ACTA1 HSPA2 G6PD
BCAR3 TUBA1C HNRNPH1
GRP HNRNPH2 HNRNPM
DBN1 GSR TUBB6
EEF1G ALB XRCC6
TRIM21 H6PD HSPA5
HSPA8 HDLBP PDIA6
EEF1A1 FUS DDX17
IGHG3 SRP72
SH3GL1 TCP1
C
A1207 JICD1
A172 JICD1
84NS
Log fraction without spheres
cells per well
siCon (0.79% GSCs)
siANXA2 (0.26% GSCs) ***
siCon (0.39% GSCs)
siANXA2 (0.08% GSCs) ***
siCon (0.34% GSCs)
siANXA2 (0.09% GSCs) ***
D
LN18 JICD1-HA-FLAG
DAPI
HA
ANXA2
20 μm
E
LN18
JICD1-HA-FLAG
IP
Input
IgG
HA
ANXA2
HA
F
HEK293T
JAG1-HA^Cterm + – +
ANXA2-FLAG – + +
Input
IP: Rabbit IgG
IP: Mouse IgG
IP: HA
IP: FLAG
HA
FLAG
G
GST-ANXA2
–
GST
GST-ANXA2
6xHis-JICD1 – + + +
Pulldown: JICD1
Input
GST
JICD1
72kDa
48kDa
35kDa
30kDa
19kDa
H
A1207
Con
JICD1
siCon
siANXA2
siCon
siANXA2
JICD1
ANXA2
β-ACTIN
19kDa
I
LN18
ANXA2
mRNA expression
siCon
siANXA2
siCon
siANXA2
Con
JICD1-HA-FLAG
J
LN18
JICD1-HA-FLAG
LN18
A1207
A1207 JICD1
JAG1 mRNA level
siCon
siANXA2

Supplementary Figure S1. Annexin A2 (ANXA2) is a JAG1 intracellular domain (JICD1)-binding protein.

(A) A graphic description of the NOTCH signaling pathway. NICD; NOTCH intracellular domain, CSL; centromere-binding protein 1/suppressor of hairless/lag-1, MSML; mastermind-like protein.

(B) A schematic presentation of JICD1-binding protein identification and a list of identified proteins.

(C) Limiting dilution sphere-forming assay performed following ANXA2 knockdown in other JICD1-expressing cell lines. ***$p < 0.001$.

(D) Microscopic images of immunofluorescence staining of HA and ANXA2 in LN18 cells expressing JICD1-HA-FLAG. Scale bar = 20 μm.

(E) Immunoprecipitation against HA in LN18 JICD1-HA-FLAG cells.

(F) Immunoprecipitation against HA and FLAG in HEK293T cells transduced with JAG1-HACterm and ANXA2-FLAG.

(G) In vitro binding assay using the purified recombinant JICD1 and GST-ANXA2. The pull-down against JICD1 in the absence of JICD1 was used as a control.

(H) Western blotting results showing that ANXA2 knockdown decreases JICD1 protein level in A1207 cells.

(I) qRT-PCR showing that ANXA2 expression is increased by JICD1 and is downregulated by ANXA2 knockdown.

(J) qRT-PCR demonstrating the mRNA level following ANXA2 knockdown in LN18 JICD1-HA-FLAG, LN18, A1207, A1207 JICD1 cells.

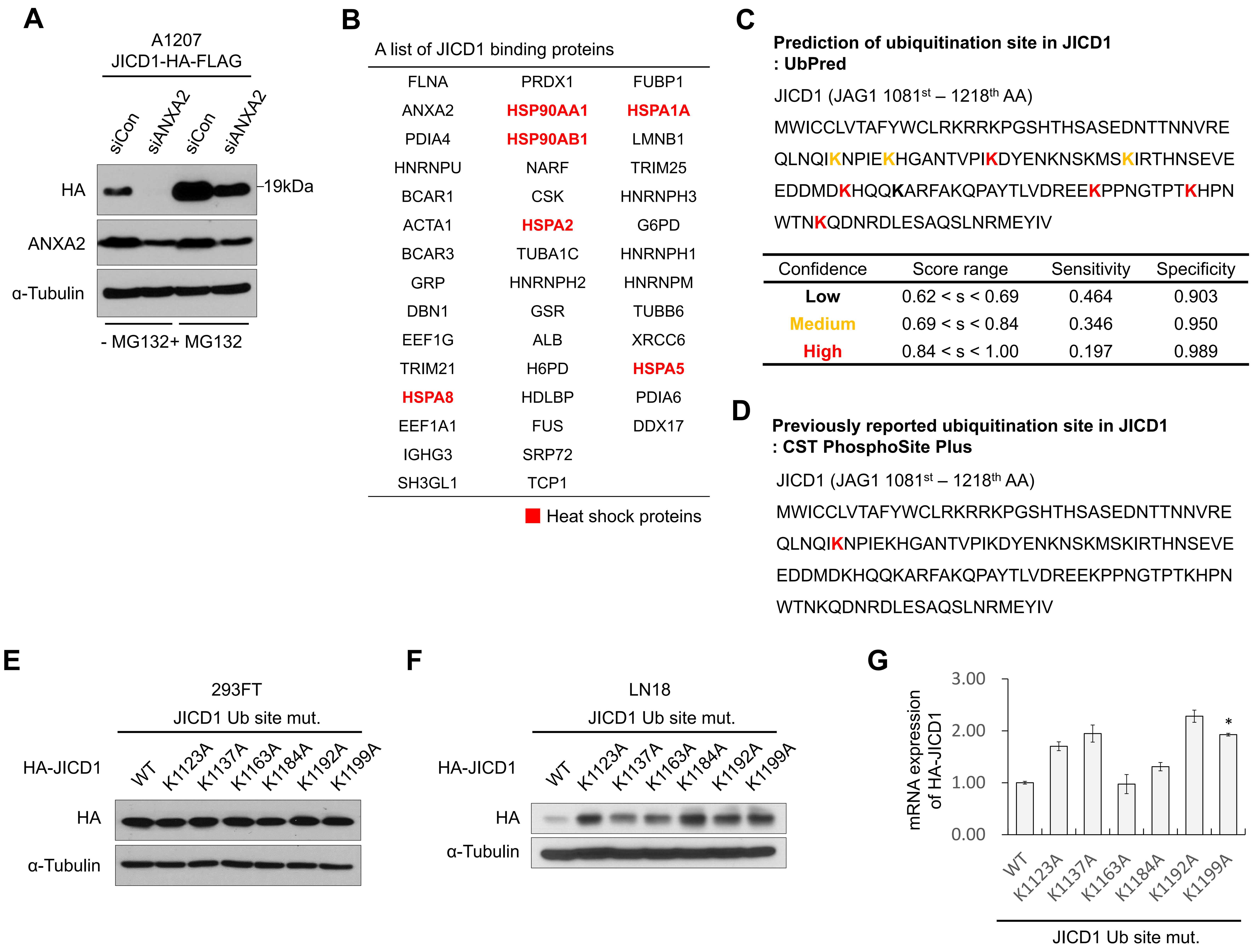

Supplementary Figure S2. The transcriptional expression of JAG1 was not changed after the ANXA2 knockdown.

(A) The protein level of JICD1-HA-FLAG following ANXA2 knockdown combined with CQ treatment in A1207-HA-FLAG cells.

(B) A list of HSP70 and HSP90 family proteins detected as JICD1-binding proteins.

(C, D) Possible ubiquitination sites in JICD1 predicted by using the (C) UbPred and (D) CST PhosphoSite Plus.

(E, F) The protein level of JICD1 with mutants in possible ubiquitination sites in (E) HEK 293T and (F) LN18 cells.

(G) Level of JICD1 mRNA with mutants in possible ubiquitination sites in LN18 cells. *$p < 0.05$.

A

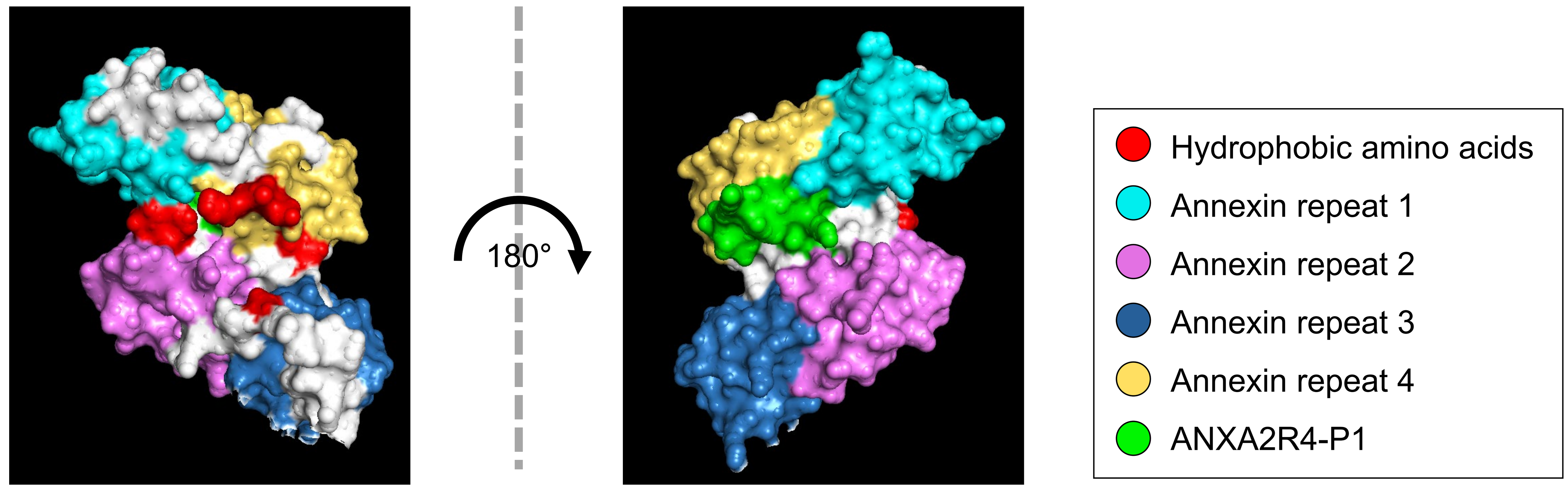

B

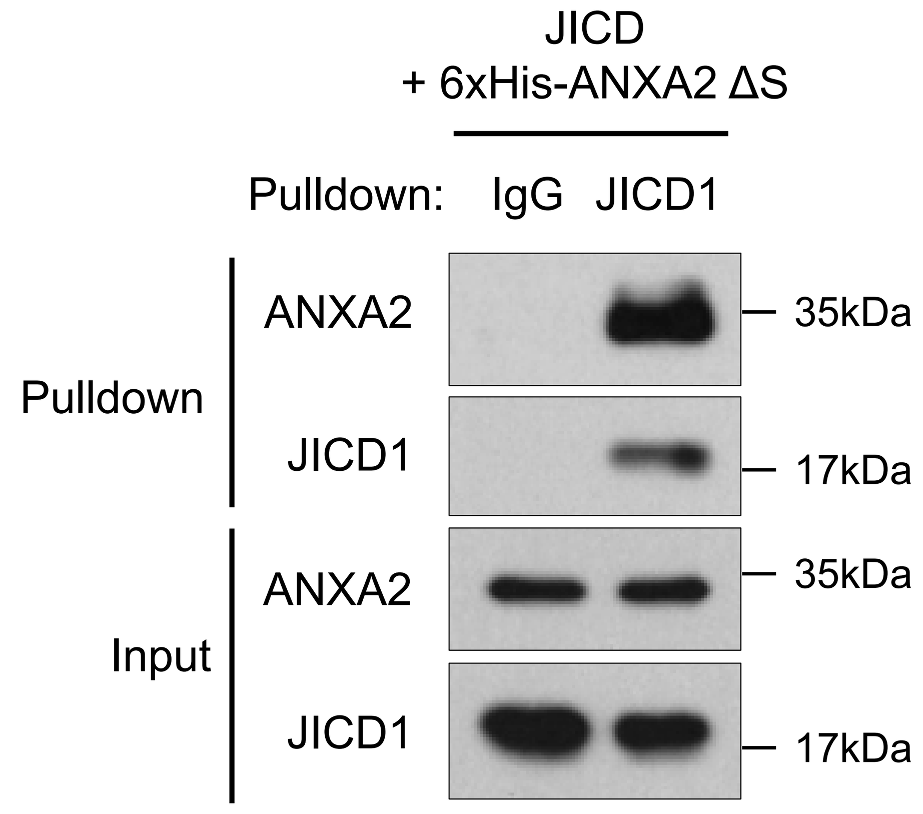

C

Peptide	Sequence	Length
R4P-1	LYDSMKGKGTRDKVLIRIMVS	21
R4P-2	RSEVDMLKIRSEFKRK	16
R4P-3	YGKSLYYYIQQ	11
R4P-4	DTKGDYQKALLYL	13

D

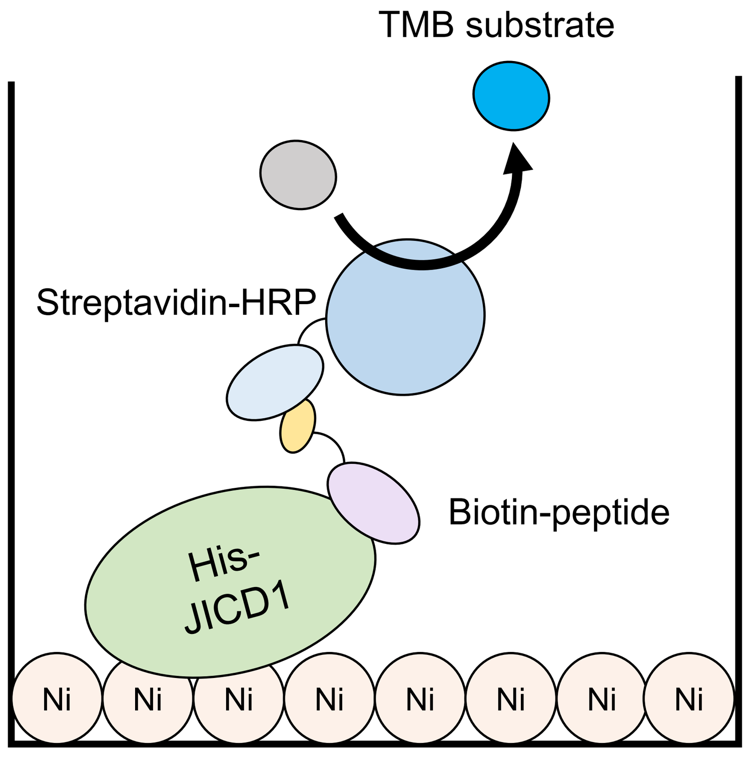

E

	Locus	Amino acid sequence	Similarity
ANXA2 R4P-1		LYD------S------MKGKGTRDK-V--LIRIMVS	
ANXA2 isoform 2	(274 – 294)	LYD------S------MKGKGTRDK-V--LIRIMVS	100(21/21)
	(109 – 123)	YDASELKAS------MKGLGT	38(8/21)
	(198 – 217)	LYD------AG-----VKRKGT-D--VPKWISIM	57(12/21)
ANXA6 isoform 1	(260 – 280)	LFK------A------MKGLGTRDN-T--LIRIMVS	76(16/21)
	(608 – 628)	LYK------S------MKGAGTDEK-T--LTRIMVS	76(16/21)
	(103 – 121)	D------A------ISGIGTDEK-C--LIEILAS	52(11/21)
	(439 – 459)	YD------AKQLKKAMEGAGTDEK-A--LI	47(10/21)
	(29 – 35)	LYT------A------MKG	24(5/21)
	(376 – 381)	MKGLGT	24(5/21)
ANXA1	(282 – 302)	LHQ------A------MKGVGTRHK-A--LIRIMVS	71(15/21)
	(127 – 143)	MKGLGTDED-T--LIEILAS	52(11/21)
ANXA4 isoform a	(256 – 276)	LYK------S------MKGLGTDDN-T--LIRVMVS	76(16/21)
	(91 – 106)	LYDVQELRRA------MKGAGT	38(8/21)
	(29 – 34)	MKGLGT	24(5/21)
ANXA5	(255 – 275)	LYY------A------MKGAGTDDH-T--LIRVMVS	71(15/21)
	(90 – 110)	LYD------AYELKHALKGAGTNEK-V--L	57(12/21)
	(28 – 33)	MKGLGT	24(5/21)
ANXA11 isoform 1	(444 – 460)	MRGAGTKDR-T--LIRIMVS	62(13/21)
	(213 – 215)	MKG	14(3/21)
ANXA8 isoform 1	(300 – 320)	LYY------A------MKGAGTRDG-T--LIRNIVS	67(14/21)
	(140 – 160)	LHD------A------MKGLGTKEG-V--IIEILAS	62(13/21)
	(30 – 36)	LYK------S------MKG	28(6/21)
ANXA13 isoform a	(254 – 273)	LYK------S------MKGAGTDEE-T--LIRIVV	67(14/21)
	(99 – 110)	MKGLGTDES-V--LI	43(9/21)
	(148 – 154)	LKKILVS	19(4/21)
	(28 – 32)	KGMGT	19(4/21)
ANXA7 isoform 1	(403 – 422)	LYY------A------MKGAGTDDS-T--LVRIVV	62(13/21)
	(239 – 259)	YD------AWSLRKAMQGAGTQER-V--LI	47(10/21)
	(176 – 178)	MKG	14(3/21)
ANXA9	(292 – 299)	V--LIRILIS	28(6/21)
	(184 – 188)	KGGRD	10(2/21)
ANXA3	(102 – 114)	S------MKGAGTNED-A--LI	38(8/21)
	(35 – 45)	GTDEK-M--LISIL	28(6/21)
	(262 – 278)	LKGIGT-DEFT--LNRIMVS	47(10/21)
	(226 – 230)	D------S------IKG	19(4/21)
ANXA10	(92 – 118)	LYD------AHELWHAMKGVGTDEN-C--LIEILAS	57(12/21)
	(267 – 275)	NK-T--VIRILI	19(4/21)

F

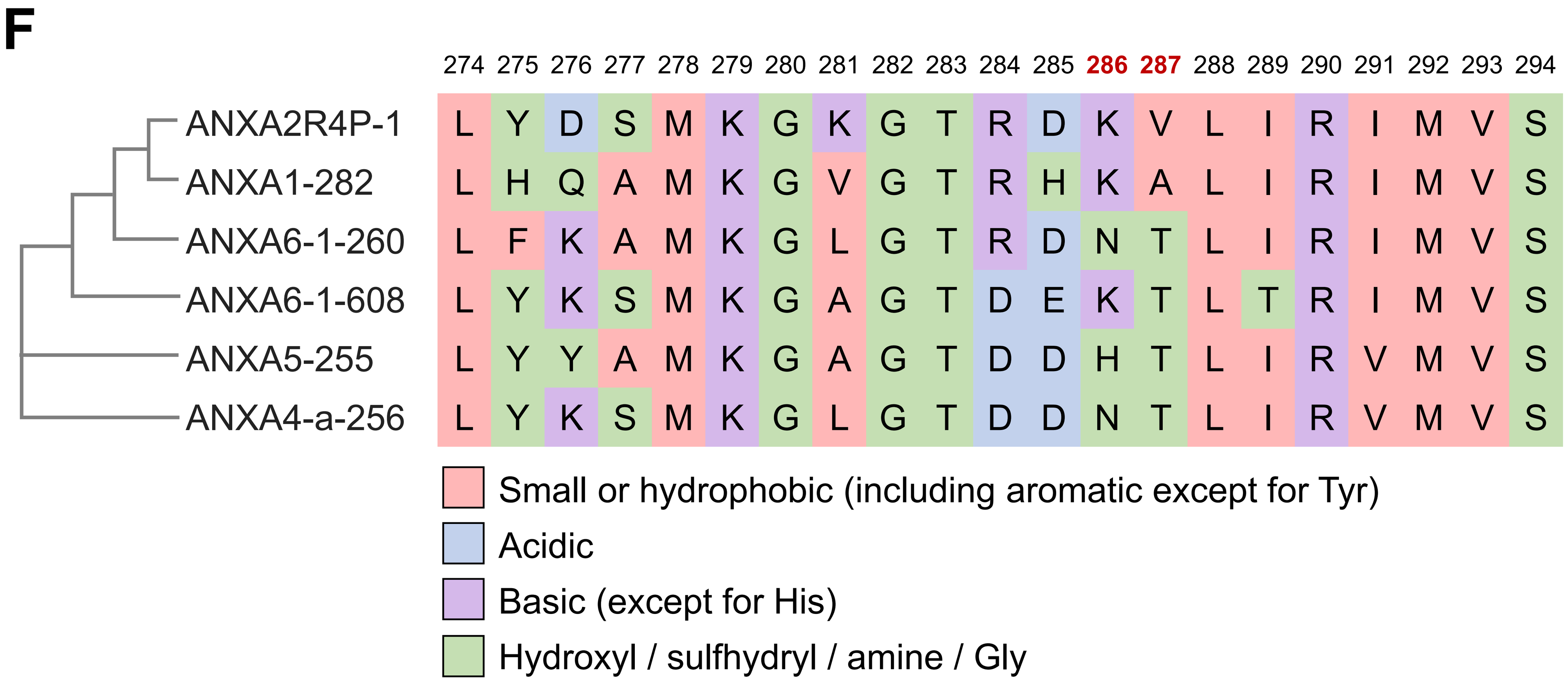

Supplementary Figure S3. R4P-1 region of ANXA2 directly binds to JICD1.

(A) Three-dimensional structural presentation of ANXA2 (PDB: 1XJL) with an indication of Annexin repeats, R4P-1.

(B) In vitro binding assay using the recombinant JICD1 and 6xHis-tagged ANXA2 lacking S domain (ANXA2ΔS).

(C) Sequences of ANXA2 R4 peptides, R4P-1, 2, 3, and 4.

(D) A graphic description of the JICD1-peptide binding assay.

(E) The result from the Basic Local Alignment Search Tool search for the proteins having a similar amino acid sequence to R4P-1.

(F) Clustering of the amino acid sequences of the peptides used in Figure S2E.

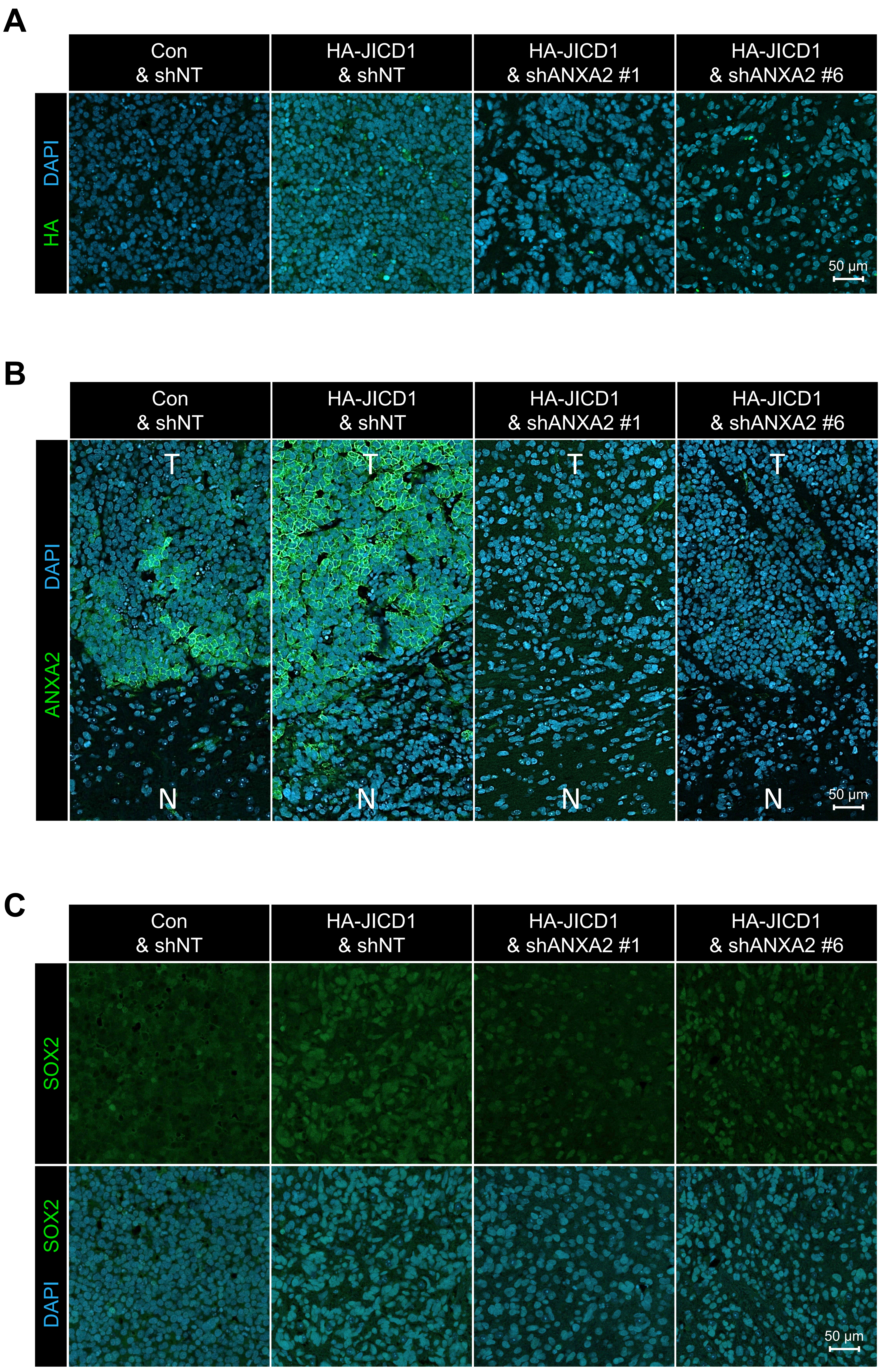

Supplementary Figure S4. Immunofluorescence images for (A) HA-JICD1, (B) ANXA2, and (C) SOX2. T: Tumor, N: Normal. Scale bar = 50 μm.

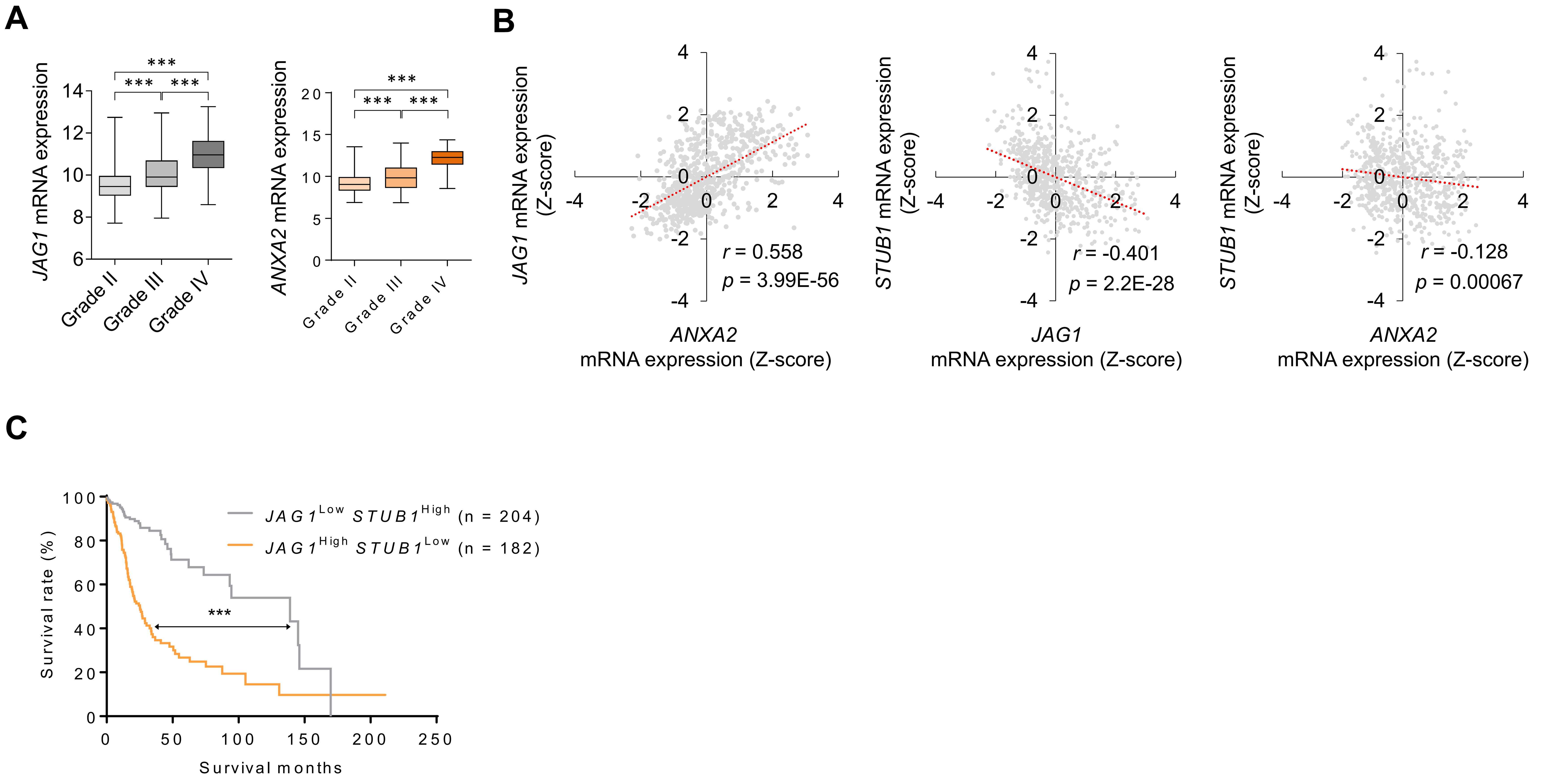

Supplementary Figure S5. JICD1–ANXA2 interaction correlates with cancer aggressiveness in patients with glioma.

(A) The mRNA expression levels of *JAG1* (left) and *ANXA2* (right) in GBM patients depending on the WHO grade. Unpaired two-tailed t-tests: $***p < 0.001$.

(B) Correlation between JAG1, ANXA2, and STUB1 in TCGA database.

(C) Survival time of patients with glioma according to expressions of ANXA2 and STUB1. The patients were divided into two groups (JAG1 high and STUB1 low versus JAG1 low and STUB1 high) based on their mRNA expression (mean ± SEM). p-value was calculated with a log-rank (Mantel-Cox) test. $***p < 0.001$.
